# Commentary: Sleep quality, quality of life, fatigue, and mental health in COVID-19 post-pandemic Türkiye: a cross-sectional study

**DOI:** 10.3389/fpubh.2024.1393054

**Published:** 2024-04-05

**Authors:** B. Sreya, Ayyagari Lakshmana Rao, Nikhil Kulshrestha, G. Ramakrishnan

**Affiliations:** ^1^Department of Commerce, SRM University, Amaravathi, Andhra Pradesh, India; ^2^Doon Business School, Dehradun, Uttarakhand, India; ^3^Homeroom Eagles, Chennai, India

**Keywords:** sleep quality, mental health, post-pandemic, public health, stress

## 1 Introduction

In this comprehensive commentary, we delve into (1) study, exploring the profound and enduring consequences of the COVID-19 pandemic on the physical and mental wellbeing of the Turkish population post-pandemic. As the global community grapples with the aftermath of this unprecedented health crisis, the study by Bener and colleagues acts as a vital lens to explore the intricate impacts on public health. Commencing in November 2019, the pandemic has left an indelible mark, with over 760 million confirmed cases and 6.9 million reported deaths worldwide by August 2023 (2). Pandemic-induced restrictions have significantly shaped the physical and mental health landscape, necessitating a closer examination of the lasting effects on individuals and communities (3). Sleep disturbances, globally linked to various medical and psychological disorders, have emerged as a prevalent concern (4), emphasizing the critical need to investigate their multifaceted effects. This study emphasizes how poor sleep quality significantly worsens overall mental health, especially in individuals with pre-existing mental health challenges, leading to elevated levels of depression, anxiety, stress, and fear symptoms. The increased prevalence of sleep disturbances during the COVID-19 pandemic is attributed to factors like the stress-laden environment, social isolation, and heightened screen time at home (5). As the ramifications of the pandemic continue to unfold, it becomes imperative to investigate and understand the predictors and associated risk factors influencing sleep quality, quality of life, fatigue, and mental health among the Turkish population in the post-COVID era. Through meticulous analysis, this commentary aims to contribute valuable insights into the enduring consequences of the pandemic, shedding light on the intricate interplay of factors shaping the physical and mental wellbeing of this population.

### 1.1 Research methodology

Bener et al. (1) conducted a comprehensive cross-sectional multi-center-based survey to explore the lasting effects of the COVID-19 pandemic on the physical and mental wellbeing of the Turkish population. The study focused on both urban and rural populations of Istanbul, with participants aged 20 years and over. Employing a robust sampling strategy, the researchers approached 3,200 individuals, achieving an 82% response rate and collecting 2,624 completed questionnaires throughout 2022. Various validated assessment tools, including WHOQOL-BREF, PHQ-15, DASS-21, GAD-7, PSQI, and FAS, were utilized to gain a nuanced understanding of quality of life, depressive symptoms, anxiety, stress, sleep quality, and fatigue. Statistical analyses in SPSS v25 involved tests for normal distribution, *t*-tests, chi-square tests, and multivariate stepwise regression analysis. By adopting a significance level of *p* < 0.01, the researchers demonstrated a stringent approach to identifying meaningful associations within the data. The inclusion of gender and age control in the regression analysis showcased a sophisticated attempt to predict potential risk factors for sleep and mental health. This methodology reflects meticulous planning, a well-calculated sample size, and a thorough sampling method. The use of validated assessment tools enhances the reliability of the findings, while statistical analyses, including multivariate regression, add depth to the exploration, laying a solid foundation for a comprehensive investigation into the enduring impact of the pandemic on the Turkish people.

### 1.2 Findings

The exploration of socio-demographic disparities in the Turkish population offers a nuanced perspective on the varied experiences within this community. The study's emphasis on gender-based differences in education, occupation, and lifestyle behaviors underscores the necessity for targeted interventions that account for the diverse factors influencing both mental health and sleep quality (6). Notably, the higher prevalence of mental health symptoms, particularly among women, highlights the urgency of adopting gender-sensitive approaches in mental health interventions (7). The intricate relationships unveiled between WHOQOL-BREF domains and sleep disorders provide valuable insights into the pervasive impact of mental wellbeing on the overall quality of life. The multivariate stepwise regression analysis serves as a key tool in identifying crucial predictors, revealing the intricate connections between mental health and sleep quality. The pinpointed factors such as depression, anxiety, stress, physical and psychological health, fatigue, and environmental elements offer a comprehensive roadmap for targeted interventions, addressing the fundamental causes of poor sleep quality (8). This insightful analysis significantly contributes to unraveling the complex interplay between socio-demographic factors, mental health, and sleep quality in the post-pandemic Turkish population. The findings broaden our understanding and offer practical implications for tailoring future public health strategies. As we navigate the aftermath of the pandemic, the importance of considering diverse factors in mental health and sleep quality interventions cannot be overstated.

## 2 Discussion

The discussion section provides a comprehensive analysis of the profound and enduring impact of the COVID-19 pandemic on the mental health and sleep quality of the Turkish population. It highlights the prevalence of mental health symptoms and sleep disturbances post-pandemic, aligning with global concerns (9). By synthesizing the study's findings with existing literature, the discussion explores the intricate association between mental health symptoms and sleep disturbances. By acknowledging study limitations, research becomes more transparent and credible. Additionally, the discussion underscores the urgency for targeted interventions to address the enduring effects of the pandemic on mental health and sleep quality, considering socio-demographic characteristics and lifestyle factors.

## 3 Conclusion

In conclusion, our analysis of Bener et al. (1) study delves into the enduring impact of the COVID-19 pandemic on the mental health and sleep quality of the Turkish population. Emphasizing the need to prioritize interventions that enhance sleep quality as a crucial aspect of public health, our findings propose potential avenues for mitigating or preventing the onset of mental health disorders. Aligned with the study's primary focus on the lasting effects of the pandemic, our conclusion underscores the imperative of addressing mental health and sleep quality within broader public health strategies (10). Despite acknowledging the study's limitations, we emphasize its significant contributions to our understanding of post-pandemic mental health challenges. In a final statement, we offer a profound commentary on the paper's future implications, highlighting its relevance in shaping targeted public health interventions in the post-pandemic era. The presented results, showcasing notable differences in socio-demographic characteristics and lifestyle factors, further accentuate the depth and breadth of the research's insights into the complexities of mental wellbeing in the aftermath of the pandemic. In essence, this conclusion stresses the critical importance of the (1) study and its potential impact on informing evidence-based practices and policies for the wellbeing of the Turkish population in the future.

## Author contributions

BS: Writing – original draft. AL: Investigation, Writing – review & editing. NK: Conceptualization, Writing – review & editing. GR: Conceptualization, Investigation, Software, Writing – review & editing.
